# Beyond COVID‐19: How the ‘dismal science’ can prepare us for the future

**DOI:** 10.1002/hec.4114

**Published:** 2020-06-02

**Authors:** Susan Chilton, Jytte Seested Nielsen, John Wildman

**Affiliations:** ^1^ Newcastle University Business School Newcastle University Newcastle upon Tyne UK

**Keywords:** COVID‐19, dismal science, future

## 1 INTRODUCTION

The actions of most governments around the globe to date to COVID‐19 bring into sharp focus the uncomfortable trade‐offs that societies face, both now and in the future, in response to a pandemic. The immediate governmental response, in most countries, of ‘managing’ the outbreak with the principal aim of ‘flattening the curve’ and ‘saving lives’ has necessitated a huge tranche of emergency fiscal support to be swiftly put in place in order to support the broader economy. For example, in the UK, the government has, by its actions, committed 20–40% (estimates vary) of GDP to managing the immediate crisis caused by the virus to support the NHS and the economy now, the full costs of which—health, fiscal and social—will be borne for many years into the future.

Attempts so far at analysing the benefits of the policy response have largely concentrated on calculating some (notional) value of ‘Lives Saved’ (the ‘policy outcome’) from current values of statistical life used in policy appraisals. For example, Greenstone and Nigam (2020) estimated a value of $60,000 per life saved to each American household. But exercises such as these are a distraction, not least given the non‐marginal nature of the policy response.

We, as a society, are beginning to realise that what we now face is effectively a ‘crisis of preparedness’. A crisis exacerbated by a lack of information regarding the spread and mortality rates of COVID‐19. However, at some point, as the crisis diminishes and the data improve, the world will take a deep breath and have to systematically consider how to deal with similar scenarios in the future. Lockdown presents an opportune time to lift our eyes, consider the *future* and think about our contribution to it as economists. How are we best placed to contribute? COVID‐19 should be seen through the lens of society, or social wellbeing, and treated as a broader public health issue.

## 2 THE ROLE OF WELFARE ECONOMICS

Welfare economics can provide a principled ex ante, proactive—as opposed to an *ex post*, reactive—framework to consider alternative policy responses or interventions going forward.

At the broadest scale, it provides us with the conceptual framework to think about the ‘big’ economy‐health trade‐offs that this crisis highlights, but it also encompasses broader social concerns such as the equity–efficiency trade‐off. Crucially, welfare economics provides us with the consideration of allocative efficiency in the widest sense, that is, an allocation of resources that maximises welfare over time and across generations, internalising as many of the externalities as possible—be they positive or negative—caused by our collective actions. At the other end of the spectrum, welfare economics provides insights into individual choices under risk and uncertainty and other behavioural factors that might prove constructive in incentivising health behaviours in the future.

The most important role of welfare economics, though, lies in its potential practical contribution to analyse ‘preparedness’ to future outbreaks of this virus or a new pandemic. To future‐proof against the very real chance of a subsequent outbreak of COVID‐19 or a new pandemic, we need to move away from the narrow perspective of the virus as primarily a ‘medical problem’, the resolution of which appears dominated by a reliance on mathematical models and behavioural science. A broader perspective might include a critical reassessment of the relationship between a government and its people and the choices and policies it (or its designated organisations) enacts on our behalf. Acknowledging the medical–social interface is not new thinking when it comes to epidemics per se, but, going forward, we will require the combined forces of many different stakeholders, for example, experts and practices from many diverse fields such as epidemiologists, health care managers, medics, and civil servants to better address the problem. From an economic perspective, all will be concerned, either implicitly or explicitly, with resource allocation.

At their simplest, economic evaluation tools can establish and highlight the benefits and costs of individual interventions but perhaps more importantly make visible the aggregate outcome of the many marginal decisions that will be made by the various stakeholders. In effect, if used with diligence, economics can provide a tool that can combine information from all relevant parties. Evaluation tools are there to aid decision makers by providing clear information that highlights the nature of the trade‐offs being made. Such tools should not replace measured discussions and other frameworks but can—and should—complement them. By being better prepared, society will almost certainly be in a more favourable position to choose the best response and understand the opportunity costs (social and health) that response involves. As well as the already noted fiscal costs, the opportunity costs will include social isolation, deepening inequalities, cancer treatments displaced, elective surgeries cancelled, and so forth.

## 3 FACING THE FUTURE

The application of the tools of economics can begin the process of building preparedness for similar future events. At one level, the lessons arising from this current scenario can be costed into future projects—what are the added benefits of having more ventilators, more nurses, more ICU beds (and more school teachers, local services, etc.) if it means we are better prepared? At a higher level, if public health experts are identifying a range of potential future interventions (e.g., contact tracing; testing; regional vs. national approaches; random sampling; antigen and antibody tests) and their impact on public health and/or medical outcomes, then economic evaluation techniques can help to make explicit the opportunity cost of these alternatives.

COVID‐19 has led to a substantial and growing number of ‘excess’ deaths in the UK. Thus, the change in mortality risk is clear. However, over time, as more becomes known about COVID‐19's effects on people, particularly across age cohorts and the potential health impacts on those who have recovered, decision makers may wish to place more weight on the impact on life expectancy and quality‐of‐life. Using willingness‐to‐pay (WTP) based approaches 1—value of a prevented fatality (VPF), value of a statistical life year (VOLY) and the value of quality‐adjusted life‐year (WTP‐QALY)—allows society to internalise the negative externalities associated with the pain, grief and suffering of death and illness. These measures allow us to analyse allocative efficiency which is the very power of the marginal analysis on which welfare economics is based; they can also add some objectivity into smaller‐scale—but just as important—decisions such as the rationing of ventilators or DNAR guidance to care homes which can be made in advance rather than, as now, in the middle of an epidemic.

We are effectively valuing the future under uncertainty when planning for new pandemics (e.g., Attema, Lugnér, & Feenstra, 2010). Costs are incurred now, the benefits later, both of which may be intergenerational. Currently, very high discount rates are being applied to future consequences, at least implicitly, without any clear rationale as to what the optimal response is. By adopting an *ex ante* approach, it may be possible to identify interventions with relatively small up‐front costs with (potentially) significant future benefits (e.g., life expectancy; lower inequalities) or, on the other hand, relatively costly interventions with low or very uncertain benefits. Arguably, past policy choices (fewer IVU beds than Germany; austerity) imply a low value (high discount rate) on the future of a potential pandemic. Would a similar policy with respect to a future pandemic reflect our preferences or not? It is incumbent upon economists to provide the answer to inform the wider debate.

## 4 CONCLUSION

We do not necessarily know what the next health crisis will be and whether it will follow a similar pattern to that displayed by COVID‐19. But we can learn lessons that may require us to re‐evaluate the benefits and costs of future programmes. The strength of our health services, the importance of our public services, our safety nets, our population health, our levels of inequality, our re‐evaluation of key workers, and so forth suddenly have taken on new importance. Smaller, extra expenditure or redistribution in the future, reflecting these benefits, may prevent larger costs further down the line, whatever the nature of the next crisis—is not that why we buy insurance?

## Footnotes

^1^
Conceptually, at least, WTP is assumed to reflect our true preferences/values. So for the purposes of this, we abstract away from the practical/ethical issues that might indeed arise if applying this frame resulted in an increase in taxes (which might happen if the benefit/cost ratio was positive for a high cost intervention).
